# A Novel Approach to Quantifying the Failure Modes of Concrete-Epoxy Interface

**DOI:** 10.3390/ma16062376

**Published:** 2023-03-16

**Authors:** Abubakar Sodiq Ishaq, Yoonju Jang, Donghyeok An, Yoseok Jeong, Ilro Youn

**Affiliations:** 1 Department of Construction and Disaster Prevention Engineering, Kyungpook National University, Sangju 37224, Republic of Korea; 2 Department of Advanced Science and Technology Convergence, Kyungpook National University, Sangju 37224, Republic of Korea; 3 Department of Computer Engineering, Changwon National University, Changwon 51140, Republic of Korea

**Keywords:** fiber-reinforced polymer, concrete-epoxy interface, fracture surface analysis, image processing, image segmentation, color space, RGB, HSV, YC_b_C_r_, CIE L*a*b*

## Abstract

The failure or debonding of CEIs (Concrete-Epoxy Interfaces) in Fiber-Reinforced Polymer concrete (FRP) systems occurs in one or a combination of three modes: CC (Cohesive failure in Concrete), CE (Cohesive failure in Epoxy), and IF (Interfacial Failure). These failure modes are usually identified, and their relationships are established by human intuition, which is prone to subjectivity. This study proposes a novel method based on image processing techniques to analyze CEI fracture surfaces and evaluate their failure modes. The failure modes of CEI fracture surfaces of specimens from a 3PB (Three-Point Bending) experiment were assessed using an HVS, CIE L*a*b*, YC_b_C_r_, or RGB color space image segmentation-based image processing technique on the preprocessed images of the CEI failure sides. A manual approach was adopted to validate the accuracy of the proposed method. Comparing the failure mode (CE) obtained using the manual and the proposed methodology, an RMSE (Root Means Square Error) of 0.19, 0.10, 0.23, and 0.26 was obtained for HVS, CIE L*a*b*, YC_b_C_r_, or RGB color space, respectively. The epoxy area selected with CIE L*a*b* color space produced the most accurate evaluation of the failure modes. This study provides an accurate method of quantifying the failure modes of CEI fracture surfaces. The methodology proposed in this study is recommended for forensic investigations to understand better the possible causes of failure in externally bounded fiber-reinforced polymers.

## 1. Introduction

Over the years since the last three decades, FRP (Fiber-Reinforced Polymer) has become the material of choice for strengthening and retrofitting aging and deteriorating infrastructures due to its relative ease of installation, high strength-to-weight ratio, resistance to corrosion, and many other advantages over traditional construction materials used for structural repairs like bolts, steel, and concrete, etc. [1,2,3,4]. FRP consists of high-strength continuous fibers embedded in a polymer matrix (resin); the embedded fibers are the reinforcement elements, while the resin serves as binders to the fiber and facilitates the transfer of loads to and within the fiber [5,6].

The polymer matrices used in the construction industry are categorized into thermosetting and thermoplastic matrices. The vinyl esters, epoxies, and polyesters are grouped under thermosetting matrices, while polypropylene, polyvinyl chloride, and polyurethane are thermoplastic matrices. Thermosetting resins are often used in FRPs for retrofitting due to their enhanced mechanical performance and high impregnation and adhesion to fibers [6,7]. CEI (Concrete Epoxy Interface) is formed during the installation of epoxy-based FRP on retrofitted or aged concrete surfaces; the CEI is the region formed at the concrete and epoxy touching surfaces [8,9,10]. In FRP concrete repair systems, the CEI is usually the region where the debonding and transition between the FRP and concrete occur [8,9,10,11,12,13,14], and the structural performance of the FRP-repaired concrete is dependent on the behavior of the CEI [8,10,13,15,16,17].

**Figure 1:**
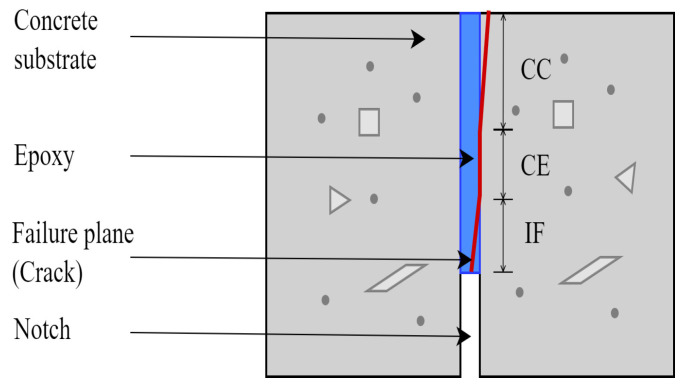
Failure modes of concrete-epoxy interface.

Over time, under sustained loading and severe environmental conditions, the structural performance of the FRP-repaired concrete weakens, subsequently fails, and debonds [4,7,8,9,10,12,13,14,15,16,18,19,20]. Failure may occur in either or combination of three ways; CC (Cohesive failure in Concrete), CE (Cohesive failure in Epoxy), and IF (Interfacial Failure), representing crack propagated along the concrete substrate, epoxy layer, and concrete epoxy interface respectively [7,8,10,13,16,20]. The CEI failure modes are depicted in Figure 1.

The CC is usually more dominant on the CEI failure surfaces than other failure types, and this is because the concrete substrate is more susceptible to failure than the FRP-epoxy bond and the concrete-epoxy interface [4,7,8,9,10,12,13,14,15,16,18,19,20]. The CE and the IF failure type ratio increases as the adhesive bond between the FRP-epoxy and concrete-epoxy degrades under sustained loading and exposure to high temperature and humidity [4,7,8,9,10,12,13,14,15,16,18,19,20,21,22,23]. Research efforts have been made toward categorizing the failure modes of CEI, yet more research needs to be made to quantify the failure modes on CEI failure surfaces. The relationships of the failure types have been based on researchers’ intuition, which is usually a product of visual inspection, as done by [9,11,12].

Attempts were made by [8,10,13] to eradicate errors influenced by human subjectivity by applying an image-processing technique to determine the ratio of failure modes. However, ref. [8,10,13] classified the failure modes based on empirical relationships. To our knowledge, there is no scientific, non-subjective, and reproducible method of quantifying the failure modes of CEI failure surfaces. This study detects and quantifies the failure modes on CEI failure surfaces using HSV (Hue, Saturation, Value), International Commission on Illumination—*Commission Internationale de l’Eclairage* (CIE) L*a*b*, YC_b_C_r_, or RGB (Red, Green, Blue) color space image segmentation-based image processing technique. The RGB color space is the primary color space and is based on color trichromatic theory, which assumes that any color can be matched by mixing the appropriate amount of the primary colors [24]. CIE L*a*b and YC_b_C_r_ color spaces are categorized under luminance chrominance color space, which expresses a color by its luminance and chromaticity. The luminance chromaticity color space was developed to improve on the limitations of the primary color space [25]. The HSV color space is a perceptual color space that mimics how humans perceive color, allowing easier communication of color between human and machine vision [24].

The approach adopted in this study classifies and quantifies the failure modes based on the local conditions of each CEI failure surface, thereby enabling accurate and economic computation of the failure modes. Precise estimation of the failure modes of the CEI failure surfaces will provide valuable insights into understanding the behavior of the CEI just before failure, the performance of the epoxy and FRP-concrete bond, and failure causes or mechanisms. Understanding the behavior of concrete-epoxy interfaces (CEI) during failure, as well as the performance of the epoxy and FRP-concrete bond, and the failure mechanisms involved, is crucial for effectively redesigning and enhancing FRP composite materials [26]. This is essential in enhancing the production of FRP composites suitable for targeted loading and environmental exposure.

## 2. Materials and Methods

This research is part of a larger research effort investigating the effects of curing time, service temperature, and sustained loading duration on the performance of FRP-concrete bonds. The focus of this study is to develop a scientific and reproducible method of quantifying the failure modes on CEI fracture surfaces. Hence, only a little information about the FRP composite, concrete, and experimental testing is provided in this study. All information regarding the test specimen, sample preparation, and experimental testing can be found in [13].

### 2.1. Mechanical Testing

A series of creep tests on 3PB (Three-Point Bending) CEI specimens were conducted by [13] to investigate the effects of epoxy curing time, sustained loading, and temperature on the performance of CEI. Below is a summary of the test specimens, test set-ups, and fracture test results.

#### 2.1.1. Test Specimen

**Figure 2:**
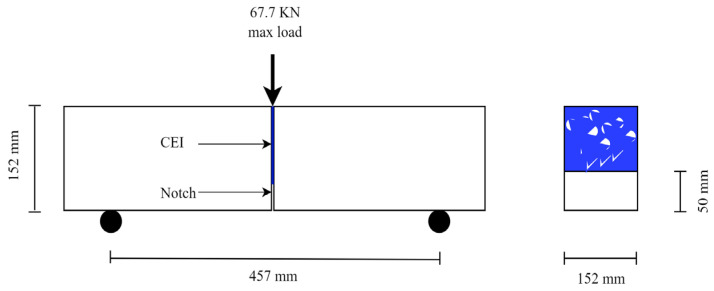
Schematic sketch of the the 3-Point Bending (3PB) experiment specimen.

Thirty-eight (38) notched 3PB test specimens were cast in a batch and cured in a tank at 23 °C and 95% relative humidity for 28 days by [13]. The geometry of the 3PB experiment specimen is shown in a schematic sketch in Figure 2. Thirty (30) of the 28-day old specimens were cut into two equal parts in the transverse direction, and the cut surfaces were then sandblasted to remove loose materials and expose the aggregates. The cut surfaces were then bonded together with epoxy. More details on the sample preparation and material properties can be found in [13]. Some specific information on the properties of the concrete and epoxy are presented in Table 1.

#### 2.1.2. Test Set-Up

**Figure 3:**
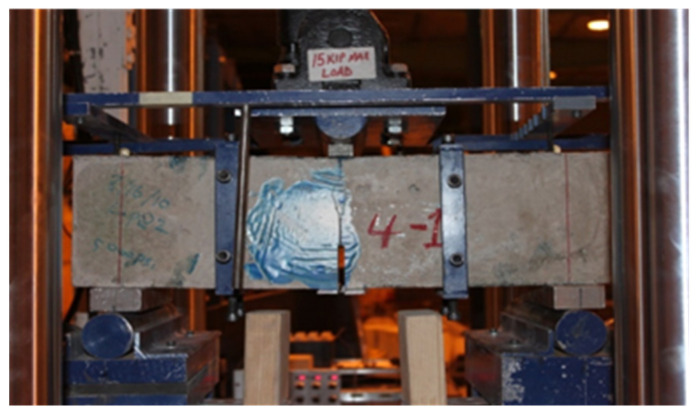
The setup of the 3PB experiment.

Figure 3 shows the test set-up for the 3PB experiment; the sustained loading was applied to the specimen using a steel frame. Details of the test set-up for the 3PB experiment can be found in [13].

#### 2.1.3. Fracture Test Results

The resulting fracture energies obtained from the 3PB experiment conducted by [13] are presented in Table 2.

The nomenclature of the specimen in Table 1 consists of three-part codes: A-B-C, where A represents the epoxy curing age before the application of load (7 or 90 days), B represents the temperature of the specimen during the sustained loading period (RT for 21 °C and HT for 30 °C), and C represents sustained loading duration (C for control, i.e., no sustained load).

### 2.2. Fracture Surface Analysis

#### 2.2.1. Fracture Surface Image Acquisition

**Figure 4:**
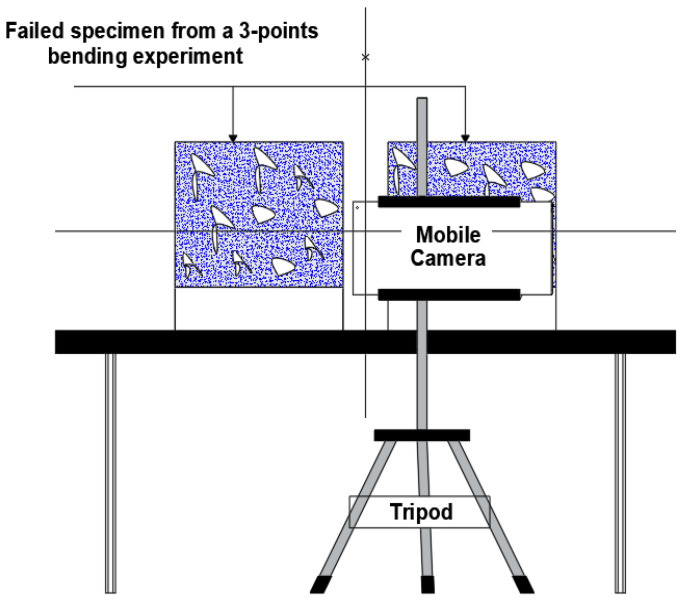
Schematic sketch of the fracture surface image acquisition set-up.

**Figure 5:**
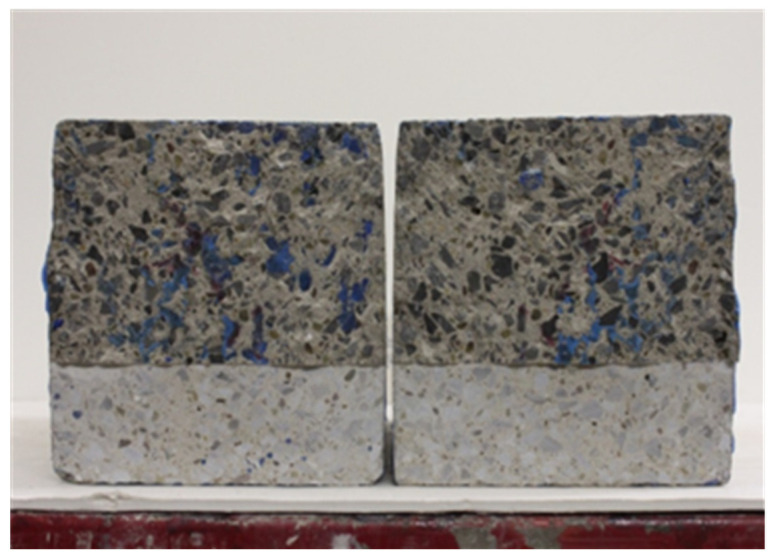
A typical image of the fracture surface failure sides.

Figure 4 shows the schematic sketch of the setup used to acquire the digital images of the CEI fracture surface (failure sides) of the 3PB experiment. Images of Twenty-eight (28) CEI fracture surfaces from the 3PB experiment were acquired using the setup. A typical digital image of the fracture surface failure sides obtained via the setup is depicted in Figure 5.

#### 2.2.2. Fracture Surface Image Preprocessing

**Figure 6:**
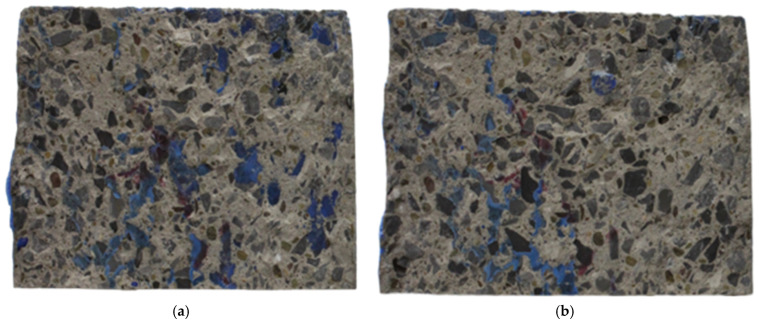
Preprocessed images of the failure side designated as (**a**) A and (**b**) B.

A commercially available graphic editor—Adobe Photoshop (version 23.1.1) by Adope Inc., San Jose, CA, USA—was used to pre-process the images of the CEI fracture surface. The quick selection tool was used to separate each of the CEI failure sides from its notch on a new canvas. Either of the failure sides was flipped horizontally. The failure sides were then points and edge mapped using the transform → skew command until corresponding points and edges were in alignment when overlaid on each other. The canvas of the two failure surfaces was then separated, and images of the two sides were saved in portable network graphic (PNG) format, such that the content of both images was visible when superimposed. Figure 6 shows the pre-processed images of the sides of the CEI failure surface of a specimen from the 3PB experiment.

#### 2.2.3. Failure Modes Detection and Quantification

After the images of the CEI failure surfaces were pre-processed, the pre-processed images were analyzed to determine and quantify the failure modes with HVS, CIE L*a*b*, YC_b_C_r_, or RGB color space image segmentation and manual approaches. The manual approach is to validate the image segmentation-based process and determine the most suitable color space for evaluating the failure modes.

- Color space image segmentation-based image processing approach

The methodology for the color spaces segmentation approach is itemized in the following steps.

Step 1: Modelling the epoxy areas on the sides of CEI fracture surfaces with a white mask using HSV, CIE L*a*b*, YC_b_C_r_, or RGB color space-based segmentation technique.

Step 2: Changing the color of the model of the epoxy areas to distinct primary color on either of the failure sides. The color of the model of sides A and B was changed to red and green, respectively.

Step 3: Superimposing the new model of sides A and B at equal intensity. That, when any area covered by either green or red color overlays, a secondary color (i.e., yellow) is formed. The regions covered in yellow imply failure within the epoxy (CE), and those covered in red and green indicate a failure within the interface of concrete substrate and epoxy (IF).

Step 4: Modelling the identified failure modes (IF and CE) with a white mask using HSV, CIE L*a*b, YC_b_C_r_ or RGB color space-based segmentation technique.

Step 5: Quantifying the failure modes. The IF, CE, and CC were evaluated with Equation (1) through (4).

**Figure 7:**
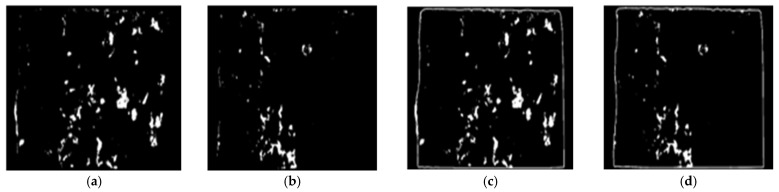
Step 1: models of epoxy areas of failure side (**a**) A (**b**) B and epoxy areas and concrete edges of (**c**) A and (**d**) B.

**Figure 8:**
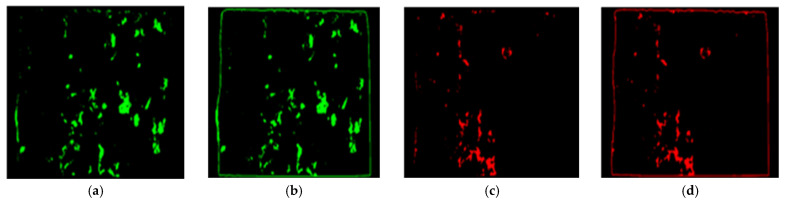
Step 2: re-modeled epoxy area with green in (**a**,**b**) for side A and red in (**c**,**d**) for side B.

**Figure 9:**
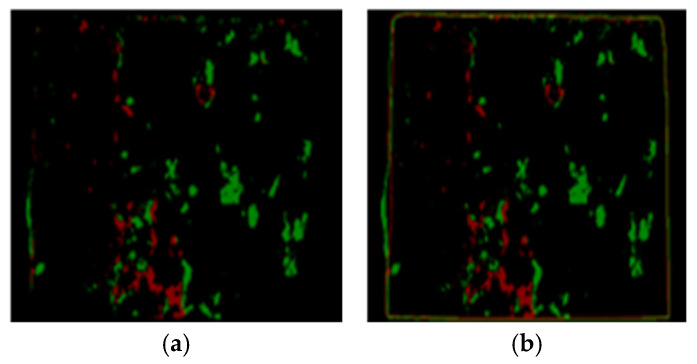
Step 3: overlapped models of the (**a**) epoxy areas and (**b**) epoxy areas and concrete edges of the failure sides A and B.

**Figure 10:**
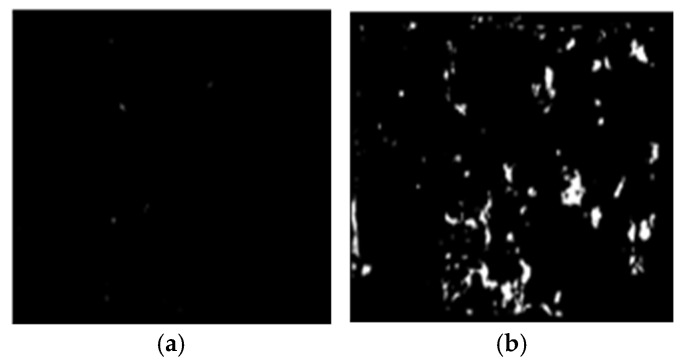
Step 4: modeling the (**a**) CE and (**b**) IF.

(1) Net area=(area covered by failure side A and B)/2,

(2) IF=(area covered by IF/net area)100,

(3) CE=(area covered by CE/net area)100,

(4) CE=( net area−[area covered by (CE+IF)]/Net area)100,

For conciseness, only the steps for the HSV color space image segmentation-based image processing technique is expressed graphically in Figure 7, Figure 8, Figure 9 and Figure 10.

The models with the concrete edges were not used in the failure mode evaluation, they are provided to monitor the overlapping of the concrete edges.

- Manual approach

The use of color space image segmentation techniques for CEI failure surface analysis is novel and there is a need to evaluate the performance of the color spaces for the proposed application. As a result, a manual or traditional approach was used to analyze the CEI failure surfaces to validate the novel image segmentation-based approach. However, due to the rigorous computational effort required by the manual approach, only one of the three failure modes was assessed. The failure surface analysis using the manual approach was limited to the evaluation of CE due to its computational ease compared to the other failure modes. The steps for evaluating the CE failure using the manual approach are itemized below.

Step 1: Printing the pre-processed images of the failed CEI surfaces on 210 mm by 297 mm (A4) papers.
Step 2: Modelling the concrete edges and epoxy areas on each CEI failure surface sides with blue and black A4-sized carbon paper, respectively. The models produced by the black and blue carbon papers were such that each represented either of the failure sides.
Step 3: Highlighting the intersection of the epoxy area on either side. As shown in Figure 7, the overlaps of the two models were highlighted in green. The green highlights imply the region where failure within the epoxy (CE) occurred.
Step 4: Evaluating CE. The ratio of the CE was evaluated with the aid of Equation (5).

(5) $$\mathrm{CE}=\left(\frac{\mathrm{Area}\;\mathrm{covered}\;\mathrm{by}\;\mathrm{yellow}\;\mathrm{highlight}}{\mathrm{Area}\;\mathrm{covered}\;\mathrm{by}\;\mathrm{the}\;\mathrm{failure}\;\mathrm{surface}}\right),$$

**Figure 11:**
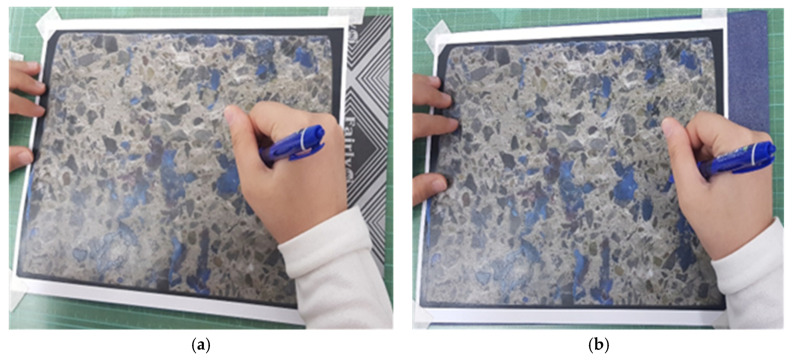
Step 2: modeling the epoxy areas and concrete edges of side (**a**) A and (**b**) B.

**Figure 12:**
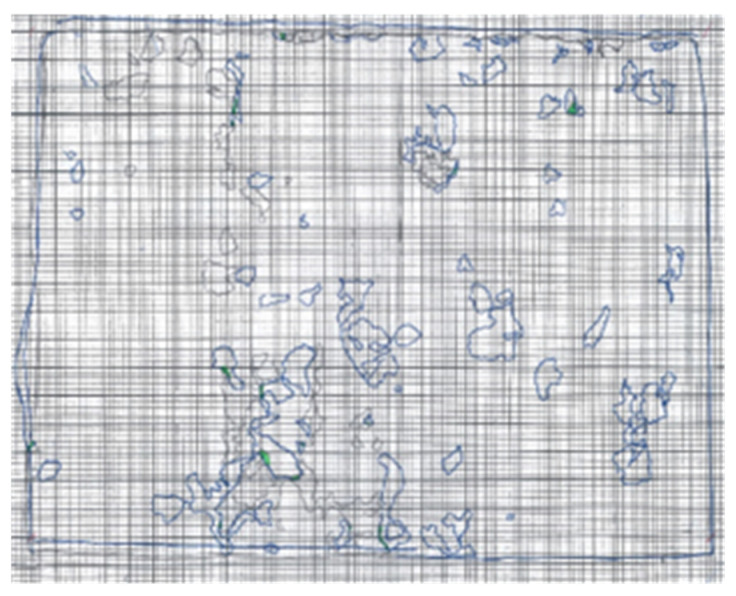
Step 3: highlighting CE in green.

The manual approach to failure surface analysis is expressed graphically in Figure 11 and Figure 12.

## 3. Results and Discussion

### 3.1. Evaluating the Performance of the Color Spaces in Modeling

#### Visual Inspection of the Models of Epoxy Areas on the CEI Failure Sides

**Figure 13:**
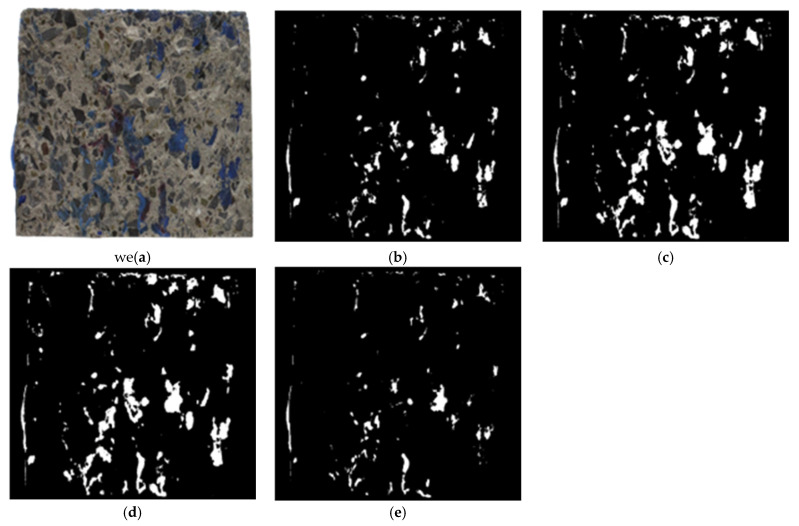
Comparison of (**a**) a CEI failure side with its epoxy stain model made with (**b**) HSV (**c**) CIE L*a*b* (**d**) YC_b_C_r_ and (**e**) RGB color space.

Image segmentation is a reliable technique for separating and simplifying images into regions with similar attributes [24,27,28,29,30]. The ideal attribute for segmenting the epoxy areas on the CEI fracture surfaces is color. Color spaces are used to segment color images. One of the core factors determining the efficacy of the techniques proposed by this study is the efficiency of the color spaces in modeling the epoxy areas on the failure sides. Figure 13 shows a typical CEI failure side and its epoxy areas models produced with HSV, CIE L*a*b*, YC_b_C_r_, or RGB color space image segmentation techniques, respectively.

As shown in Figure 13, the model of the epoxy areas on the CEI failure side differs across the color spaces. This is because the accuracy of the color spaces in interpreting and separating colors varies [24,25,29,30]. RGB color space cannot separate color intensities [24]. HSV color space is suitable for separating colors in images with uniform backgrounds [30]. At the same time, the YC_b_C_r_ is a television color space [24] and is suitable for separating colors in images with uneven backgrounds and illumination [30]. Of all color spaces, CIE-based color spaces like CIE L*a*b* have the highest sensitivity to slight differences among colors due to their ability to represent any color with very high precision [31]. A comparison of the epoxy areas models shown in Figure 13 shows that the models produced by CIE L*a*b color space best represent the epoxy areas on the CEI failure sides. The performance of HSV color space is almost as good as CIE L*a*b, while RGB color space produces the worst models. This is because both CIE L*a*b and HSV color spaces mimic the human perception of color; RGB color space is unsuitable for color-based detection due to its inability to separate color intensities [24,25,29].

### 3.2. Disparity of the CE Estimated with the Image Segmentation and the Manual Approaches

The HSV, CIE L*a*b*, YC_b_C_r_, or RGB color spaces image segmentation approach estimated varying CC, CE, and IF values for the same CEI failure surface. The difference in the CEI failure modes evaluated by the color spaces-based image segmentation approach is due to the disparity in how each color space interprets colors [24,25]. Table 3 shows the CEs estimated using the HSV, CIE L*a*b*, YC_b_C_r_, and RGB color space image segmentation-based and manual approaches.

The accuracy of each of the color spaces in detecting and quantifying the failure modes (IF, CE, and CE) was determined by measuring the disparity between CEs obtained from the manual and the respective color spaces image segmentation-based approach. RMSEs (Root Means Square Errors) of 0.19, 0.10, 0.23, and 0.26 was obtained when the CEs evaluated by the manual approach were compared with the CEs evaluated by the HVS, CIE L*a*b*, YC_b_C_r_, or RGB color space-based image segmentation approach, respectively.

**Figure 14:**
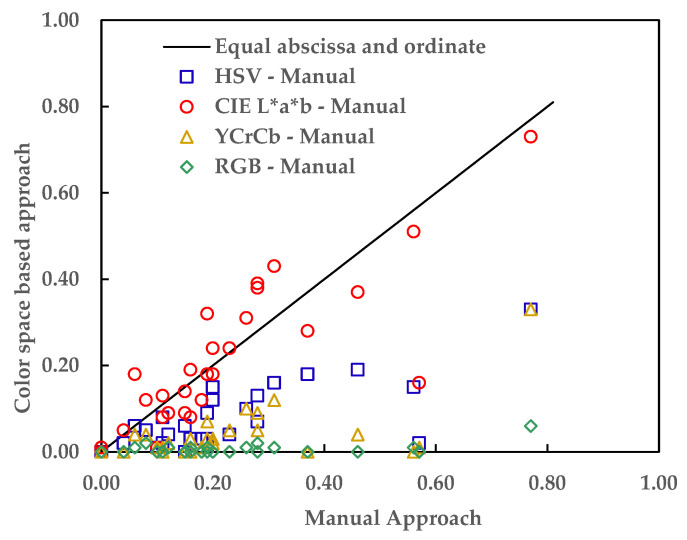
Performance evaluation of the color spaces.

Figure 14 shows the disparity between the CEs obtained from the color spaces-based image segmentation and the manual approaches. As shown in Figure 14, the CIE L*a*b* is the best-performing color space with a root mean square error of 0.10.

### 3.3. Analysis of the Correlations between the Evaluated Failure Modes and the Performance of the Epoxy Bond

Table 4 shows the ratio of the failure mode on the CEI failure surfaces; the highest (at least 81%) and the least (at most 1%) estimated failure modes are CC and CE, respectively, across the color spaces. This implies that the reinforced polymer bond is more robust and less susceptible to failure than the concrete-concrete cohesive bond and the concrete-epoxy adhesive bond [4,7,8,9,10,12,13,14,15,16,18,19,20].

**Figure 15:**
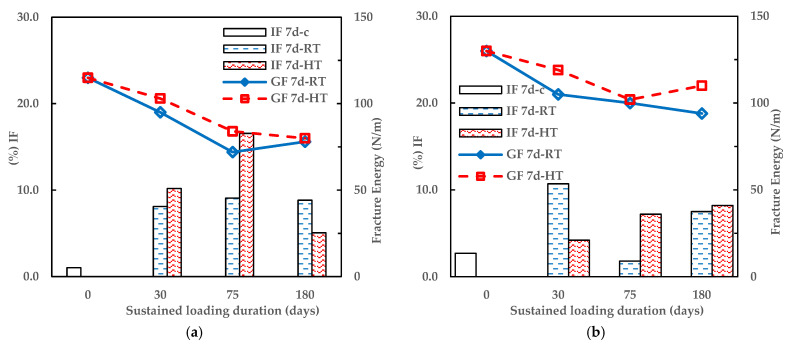
Percentage of IF failure modes (determined using CIE L*a*b* color-space) of 3PB and fracture energy against Sustained loading duration (**a**) 7-days and (**b**) 90 days cured specimen.

As shown in Figure 15, as the IF increases, the fracture energy reduces as the temperature and humidity conditions of the climate-controlled chamber change from 30% relative humidity at 21 °C (RT) to 25% relative humidity at 30 °C (HT) and as the sustained loading period (days) increases. This confirms the inverse relationship between the strength of physical infrastructure (in this case, CEI bond), environmental loads, and sustained loading duration [4,8,10,13,19,32,33].

## 4. Conclusions

This study has presented a novel approach based on image processing to quantify the failure modes (cohesive failure in epoxy (CC), cohesive failure in epoxy (CE), and interfacial failure (IF)) on concrete-epoxy interface failure surfaces. This study evaluated the quantity of the failure modes of twenty-eight (28) CEI failure surfaces specimens from the 3PB experiment conducted by [13] using four significant steps: image acquisition, image pre-processing, epoxy area selection, and failure mode detection and quantification. Based on fracture surface analysis, the following conclusions can be drawn:

1. The epoxy area and the evaluated failure modes vary across the color spaces. RMSEs (Root Means Square Errors) of 0.19, 0.10, 0.23, and 0.26 was obtained when the CEs evaluated by the manual approach were compared with the CEs evaluated by the HVS, CIE L*a*b*, YC_b_C_r_, or RGB color space-based image segmentation approach, respectively.
2. The CIE L*a*b* is the ideal color space for the novel approach proposed in this study, as it produced the most accurate model of the epoxy areas on the failure side and the closest evaluation of the failure mode (CE) to those made by the manual approach.
3. Analysis of the evaluated failure modes shows that the failure modes have some correlations with the bond performance. The agreement of the correlations with established literature further implies the suitability of the novel approach for quantifying the failure modes of the concrete-epoxy failure surfaces.
4. The epoxy matrix and the concrete substrate are the least and most susceptible to failure, respectively. The performance of the interfacial bond between the concrete and the epoxy weakens as environmental loads and sustained loading duration increase.
5. The novel approach proposed in this study provides a scientific, non-subjective, and reproducible technique to quantify the failure mode on concrete-epoxy interface fracture surfaces in externally bonded FRP-repaired concrete, provided that both concrete and epoxy have distinctive colors. The novel approach proposed in this study is perfect for forensic engineering as it can give valuable insight into the probable causes of failure.

## Figures and Tables

**Table 1:** Materials Properties.

Material Properties	Values
28 days Compressive Strength of concrete (MPa)	34.5
Tensile strength of concrete (MPa)	2.85
Elastic modulus of concrete (GPa)	27.7
Elastic modulus of epoxy (GPa)	3.10
Yield strain of epoxy (%)	0.4
Tensile strength of epoxy (GPa)	55.2

Source [13].

**Table 2:** Total Fracture Energy for the 3PB.

Specimen ID	Sustained Loading Duration (Days)	Average Fracture Energy (N/m)
7d-C	-	115
7d-HT-SL30d	32	103
7d-RT-SL30d	31	95
7d-HT-SL75d	77	84
7d-RT-SL75d	77	72
7d-HT-SL180d	181	80
7d-RT-SL180d	180	78
90d-C	-	130
90d-HT-SL30d	32	119
90d-RT-SL30d	31	105
90d-HT-SL75d	75	102
90d-RT-SL75d	76	100
90d-HT-SL180d	182	110

Source [13].

**Table 3:** CE estimated using manual and color space image segmentation approaches.

Specimen	HSV	CIE L*a*b*	YC_b_C_r_	RGB	Manual
7d-C-1	0.00	0.00	0.00	0.00	0.00
7d-C-2	0.04	0.09	0.02	0.01	0.12
7d-HT-SL30d-1	0.00	0.09	0.00	0.00	0.15
7d-HT-SL30d-2	0.13	0.38	0.09	0.02	0.28
7d-RT-SL30d-1	0.03	0.19	0.03	0.01	0.16
7d-RT-SL30d-2	0.15	0.51	0.00	0.01	0.56
7d-HT-SL75d-1	0.04	0.24	0.05	0.00	0.23
7d-HT-SL75d-2	0.01	0.01	0.01	0.00	0.10
7d-RT-SL75d-1	0.07	0.39	0.05	0.00	0.28
7d-RT-SL75d-2	0.06	0.18	0.04	0.01	0.06
7d-HT-SL180d-1	0.19	0.37	0.04	0.00	0.46
7d-HT-SL180d-2	0.08	0.13	0.01	0.00	0.11
7d-RT-SL180d-1	0.03	0.08	0.00	0.00	0.16
7d-RT-SL180d-2	0.15	0.24	0.03	0.00	0.20
90d-C-1	0.00	0.01	0.00	0.00	0.00
90d-C-2	0.06	0.14	0.00	0.00	0.15
90d-HT-SL30d-1	0.33	0.73	0.33	0.06	0.77
90d-HT-SL30d-2	0.09	0.32	0.07	0.01	0.19
90d-RT-SL30d-1	0.02	0.05	0.00	0.00	0.04
90d-RT-SL30d-2	0.05	0.12	0.04	0.02	0.08
90d-HT-SL75d-1	0.12	0.18	0.02	0.00	0.20
90d-HT-SL75d-2	0.18	0.28	0.00	0.00	0.37
90d-RT-SL75d-1	0.16	0.43	0.12	0.01	0.31
90d-RT-SL75d-2	0.10	0.31	0.10	0.01	0.26
90d-HT-SL180d -1	0.02	0.08	0.00	0.00	0.11
90d-HT-SL180d -2	0.03	0.18	0.03	0.00	0.19
90d-RT-SL180d -1	0.03	0.12	0.01	0.00	0.18
90d-RT-SL180d -2	0.02	0.16	0.01	0.00	0.57

**Table 4:** The Ratio of the Failure Modes on the CEI Fracture Surfaces.

Specimen	HSV (%)			CIE L*a*b (%)			YC_b_C_r_ (%)			RGB (%)		
	CE	IF	CC	CE	IF	CC	CE	IF	CC	CE	IF	CC
7d-C-1	0	0.31	99.69	0	0.74	99.26	0	0.06	99.94	0	0.01	99.99
7d-C-2	0.04	1.19	98.77	0.09	2.21	97.7	0.02	0.61	99.37	0.01	0.26	99.73
7d-HT-SL30d-1	0	2.07	97.93	0.09	5.07	94.03	0	1.05	98.95	0	0.29	99.71
7d-HT-SL30d-2	0.13	6.3	93.57	0.38	10.74	88.88	0.09	3.97	95.94	0.02	1.85	98.13
7d-RT-SL30d-1	0.03	6.58	93.39	0.19	10.06	89.75	0.03	2.93	97.04	0.01	1.16	98.83
7d-RT-SL30d-2	0.15	5.98	93.86	0.51	10.32	89.16	0	2.89	97.11	0.01	1.67	98.32
7d-HT-SL75d-1	0.04	6.78	93.18	0.24	12.93	86.83	0.05	4.88	95.07	0	1.53	98.47
7d-HT-SL75d-2	0.01	1.57	98.43	0.01	5.3	94.68	0.01	1.1	98.89	0	0.25	99.75
7d-RT-SL75d-1	0.07	9.86	90.07	0.39	17.74	81.87	0.05	4.78	95.17	0	2.38	97.62
7d-RT-SL75d-2	0.06	11.07	88.87	0.18	15.37	84.45	0.04	7.86	92.09	0.01	5.29	94.7
7d-HT-SL180d-1	0.19	10.69	89.12	0.37	13.89	85.74	0.04	3.34	96.62	0	0.25	99.75
7d-HT-SL180d-2	0.08	3.33	96.59	0.13	3.79	96.08	0.01	0.56	99.43	0	0	100
7d-RT-SL180d-1	0.03	4.51	95.46	0.08	5.49	94.43	0	1.32	98.68	0	0.04	99.96
7d-RT-SL180d-2	0.15	3.88	95.97	0.24	4.64	95.12	0.03	1.49	98.48	0	0.06	99.94
90d-C-1	0	0.44	99.56	0.01	1.43	98.56	0	0.44	99.56	0	0.26	99.74
90d-C-2	0.06	1.9	98.04	0.14	2.73	97.13	0	0.44	99.56	0	0.11	99.89
90d-HT-SL30d-1	0.33	8.95	90.71	0.73	12.82	86.45	0.33	7.96	91.71	0.06	4.15	95.79
90d-HT-SL30d-2	0.09	3.89	96.02	0.32	8.51	91.17	0.07	3.02	96.91	0.01	0.87	99.11
90d-RT-SL30d-1	0.02	2.88	97.1	0.05	3.5	96.44	0	1.44	98.56	0	0.43	99.57
90d-RT-SL30d-2	0.05	3.83	96.11	0.12	4.81	95.07	0.04	2.98	96.98	0.02	1.81	98.17
90d-HT-SL75d-1	0.12	2.11	97.76	0.18	2.87	96.95	0.02	0.61	99.38	0	0.15	99.85
90d-HT-SL75d-2	0.18	0.61	99.2	0.28	0.69	99.03	0	0.05	99.95	0	0	100
90d-RT-SL75d-1	0.16	8.6	91.24	0.43	11.93	87.64	0.12	6.08	93.81	0.01	2.96	97.02
90d-RT-SL75d-2	0.1	1.69	98.22	0.31	2.51	97.18	0.1	0.79	99.12	0.01	0.32	99.67
90d-HT-SL180d -1	0.02	4.02	95.95	0.08	5.45	94.47	0	1.84	98.16	0	0.57	99.43
90d-HT-SL180d -2	0.03	6.75	93.22	0.18	9.62	90.2	0.03	5.39	94.58	0	2.39	97.61
90d-RT-SL180d -1	0.03	5.72	94.25	0.12	8.09	91.79	0.01	2.76	97.23	0	0.92	99.08
90d-RT-SL180d -2	0.02	5.04	94.93	0.16	8.3	91.54	0.01	4.6	95.39	0	2.37	97.63

## Data Availability

Not applicable.
